# Supplementary material for: The Mechanisms of Action of Hyperbaric Oxygen in Restoring Host Homeostasis during Sepsis
Source: Biomolecules. 2023 Aug 7;13(8):1228. doi: 10.3390/biom13081228 (PMC10452474; doi:10.3390/biom13081228)
Supplement: Supplementary file 1 [file biomolecules-13-01228-s001.zip › biomolecules-2540691-supplementary.pdf]

Table S1 - Studies on the effect of hyperbaric oxygen treatment on HIF-1α

Study	Method	Outcome	O_2 environment	Disease under study	No. of objects exposed to HBO_2	Specimen	HBO treatment
Capo et al. 2023	ELISA	HIF-1α was increased after HBOT, but reduced 28 days after wound healing	Relative hypoxic, chronic inflammation	Chronic diabetic wound	18	In vivo study of human peripheral blood	2.2 ATA 20 treatments, 5/week 1 hour/day
Xue et al. 2023	Western blot ELISA	HBOT, at both 1.5 and 2.5 ATA, reduced the increased levels of HIF-1α in both brain tissue and serum.	Hypoxic, acute inflammation	Ischemic brain injury	24	In vivo rat model, Brain tissue and serum levels	1.5 or 2.5 ATA 1 hour 6 days
Syahrizal et al. 2022	Immunohisto chemistry	Protein expression of HIF-1α lower in HBO_2 group compared to inflamed and healthy controls.	Relative hypoxic, chronic inflammation	Endometriosi s	14	In vivo mouse model	2.4 ATA 10 days 1,5 hours/day, incl 2 airbreaks
Arienti et al. 2021	Real time-quantitative PCR and Western blot	HBO_2 decreased disease induced elevated levels of HIF-1α protein and mRNA expression.	Hypoxic, chronic inflammation	Human glioblastoma	10	Ex vivo study of human fresh tumor tissue	2.5 ATA 1 hour
Chen et al. 2021	Immuno-fluorescence and Western blot	HBO_2 decreased injury induced elevated levels of HIF-1α protein expression. Levels remained higher than health controls.	Relative hypoxic, chronic inflammation	Burn injury	6	In vivo rat model	2.5 ATA 14 days 1,5 hours/day
Fratantonio et al. 2021	Western blot	HBO_2 did not significantly change HIF-1α protein expression.	Normoxic, non-inflammation	Healthy	4	In vivo study of human peripheral blood mononuclear cells	~1.4 ATA 1 hour
Wang et al. 2021	Immunostaini ng and western blot	HBO_2 decreased HIF-1α protein expression compared to injured controls. Levels remained higher than healthy controls.	Relative hypoxic, acute inflammation	Cerebral ischemia reperfusion injury	8	In vivo rat model	1.5 ATA 3 days 1,5 hours/day
Yuan et al 2021	Western blot	HBO_2 reduced the disease induced HIF-1α protein expression compared to controls.	Relative hypoxic, acute inflammation	Pulmonary fibrosis	3	Ex vivo study of human lung fibroblast	2.5 ATA 14 days 1,5 hours/day
Zhang et al 2021	Quantitative PCR analysis of the HIF-1α target genes	HBO_2 downregulated cancer induced HIF-1α signaling.	Hypoxic, acute inflammation	Non-small-cell-lung cancer	6	Ex vivo study of hypoxic cell lines	3 ATA 1,5 hours
Harnanik et al. 2020 (Infect Dis Rep)	Immuno-histo-chemistry	HBO_2 decreased HIF-1α protein expression compared to controls.	Relative hypoxic, chronic inflammation	Rheumatoid arthritis	16	In vivo mouse model	2.4 ATA 10 days 1,5 hours/day, incl 2 airbreaks

Harnanik et al. 2020 (Oman Med J)	Immuno-histo-chemistry	HIF-1α protein expression decrease in response to HBO_2 in both treatment schedules compared to controls.	Relative hypoxia, acute inflammation	Model of rheumatoid arthritis	16	In vivo mouse model	2.4 ATA 10 or 5+5 days 1,5 hours/day, incl 2 airbreaks
Huang et al. 2020	Quantitative-PCR and Western blotting.	HIF-1α mRNA and protein level increased progressively in response to HBO_2 compared to diseased controls.	Normoxic, acute inflammation	Diabetic model	3	Ex vivo human skin fibroblast	2.5 ATA 1, 2 or 3 days 1,5 hours/day
Huang et al. 2020	Quantitative-PCR and Western blotting.	HIF-1α mRNA and protein level increased in response to HBO_2 compared to diseased controls.	Relative hypoxic, chronic inflammation	Diabetic foot animal model	3	In vivo mouse model	2.0 ATA 30 days 1 hour/day
Mihaljevic et al. 2020	Real-time quantitative PCR and Western blot	Expression of HIF-1α mRNA and proteins levels were significantly increased in response to HBO_2 at both time points compared to untreated controls	Normoxic, non-inflamed aortic tissue	Vascular reactivity	6 (PCR) 8 (western blot)	Ex vivo aortic rings from rats	2.0 ATA After 24 hours and 4 days 2 hours/day
Salmon-Gonzales et al. 2020	Real time-Quantitative-PCR	HBO_2 did not affect HIF-1α mRNA expression.	Normoxic, non-inflammatory	Bone metabolism	Not specified	Ex vivo study of human osteoblast cell line	2.3 ATA 1, 3 or 5 days 1,5 hours/day
Yamamoto et al. 2020	Real time-quantitative-PCR, ELISA	HBO_2 did not affect HIF-1α mRNA levels, but increased HIF-1α protein expression compared to injured controls.	Relative hypoxic, acute inflammation	Muscle regeneration after contusion	6	In vivo rat model	2.5 ATA 5 days 2 hours/day
Utami et al. 2020	Immunohisto chemistry	HBO_2 decreased HIF-1α protein expression compared to disease induced controls. Levels remained higher than healthy controls.	Relative hypoxic, acute inflammation	Cerebral malaria	13	In vivo mouse model	2.4 ATA 10 days 1,5 hours/day, incl 2 airbreaks
Anguiano-Hernandez et al 2019	Immunohisto chemistry	HBO_2 did not change HIF-1α protein expression compared to before HBO_2. No control group.	Relative hypoxic, chronic inflammation	Diabetic foot ulcers	18	In vivo study of human wound tissue	1.4 ATA 20 days, 5/week 1 x 45 min/day
Ergozen et al. 2019	PCR	HBO_2 treatment increased HIF-1α mRNA expression compared to before intervention.	Relative hypoxic, chronic inflammation	Diabetic ulcers	20	In vivo study of human skin biopsies	2.5 ATA 10 days, 5/week 1 x 85 min/day, incl 2 airbreaks
Harnanik et al. 2019	Immunohisto chemistry	HBO_2 decreased HIF-1α protein expression compared to disease induced controls.	Relative hypoxic, chronic inflammation	Rheumatoid arthritis	10	In vivo mouse model	1.7 ATA 10 days 1,5 hours/day, incl 2 airbreaks
Hsu et al. 2019	Immunofluor escence and western blot	HBO_2 increased HIF-1α protein expression compared to healthy and ischemic controls evaluated 9 days after intervention.	Relative hypoxic, chronic inflammation	Critical-limb ischemia	8	In vivo rat model	2.4 ATA 5 days 3 hours/day

Lin et al. 2018	ELISA	HIF-1α protein expression decreased progressively from 1 to 3 treatments.	Hypoxic, chronic inflammation	Peripheral arterioclerotic arterial occlusive disease	57 for each time interval	In vivo study of human blood	2.5 ATA 1, 2 or 3 days 2 hours/day
Song et al. 2018	Immunohisto chemistry	HIF-1α protein expression decrease in response to HBO_2 compared to controls	Relative hypoxic, chronic inflammation	Keloid	8	In vivo study of human tissue	2.0 ATA 20 days or more 2 hours/day
Zembrzuska et al. 2019	ELISA	HBO_2 increases HIF-1α protein expression levels compared to disease induced controls.	Normoxic, chronic inflammation	Glioblastoma	Not specified	Ex vivo study of human glioblastoma cells	2.0 ATA 1 hour
Zhang et al. 2018	Real time-quantitative - PCR, immunoflures cence staining and western blotting	HBO_2 decreased HIF-1α protein expression compared to diseased controls. Levels remained higher than healthy controls.	Relative hypoxic, chronic inflammation	Keloid scars	9	In vivo study of human tissue	2.0 ATA 7 days 1 hour/day
Ravaioli et al 2018	Gene expression analysis and gene mapping	HBO_2 decreased HIF-1α mRNA expression (non-significant) compared to untreated controls.	Hypoxic, non-inflammation	Kidney transplantatio n	4	(Ex vivo) Post mortem donor kidneys	1.5 ATA 1 HBO session Duration not specified
Li et al. 2017	Western blot	HBO_2 decreased injury-induced elevated levels of HIF-1α protein expression. Levels remained higher than healthy controls.	Relative hypoxic, chronic inflammation	Arteriovenou s fistula	32 (sham) + 32 (diseased)	In vivo rabbit model	2.5 ATA 20 days 1 hour/day
Gang Wang et al. 2017	Immunohisto chemistry	HBO_2 decreased HIF-1α protein expression compared to controls.	Relative hypoxic, acute inflammation	Periodontitis	12	In vivo rat at model	2.5 ATA 14 days 1 hour/day
Novak et al 2016	Real-Time PCR	HBO_2 increased HIF-1α mRNA expression when comparing to both diseased and healthy controls. No effect was observed in healthy controls.	Relative hypoxic, acute inflammation	Model of induced colitis	4	In vivo mouse model	2.4 ATA 15 days 2 x 1 hour/day
Lu et al. 2016	Immunohisto chemistry and western blot	HIF-1α protein expression decreased in response to HBO_2 in both chemotherapy treated and untreated glioma mice compared to their respective controls.	Relative hypoxic, chronic inflammation	Healthy mice inoculated with human glioma cells	5 (glioma mice) and 5 (Nimustine treated glioma mice)	In vivo mouse model	2.5 ATA 21 days 1,5 hours/day
Ding et al. 2015	Immunohisto chemistry	HBO_2 increased HIF-1α protein expression compared to controls.	Relative hypoxic, acute inflammation	Intracranial glioma	5	In vivo rat model	3.0 ATA 12 days 1 hour every second day
Parra et al 2015	Immunohisto chemistry	HBO_2 reduced disease increased HIF-1α protein expression compared to healthy controls.	Hypoxic, acute inflammation	Animal models with or without colitis	7 (colitis) 7 (healthy)	In vivo rat model	2,0 ATA 2 days 2 hours/day
Zhou et al. 2015	Western blot	HBO_2 reduced the injury induced HIF-1α protein expression compared to controls.	Hypoxic, acute inflammation	Intracerebral hemorrhage	6	In vivo mouse model	3.0 ATA 1 hour

Hu et al. 2014	Western plot and ELISA	Delayed HBO_2 significantly increased HIF-1α protein expression compared to injured and healthy controls.	Normoxic, chronic inflammation	Late-chronic phase of stroke	7	In vivo rat model	2.5 ATA 7 days 1,5 hours/day
Yildirim et al. 2014	Unspecified (routine biochemistry)	HBO_2 decreased HIF-1α protein expression in soft tissue after first treatment session compared to injured controls, but the difference was not significant after 2 sessions. Plasma levels of HIF-1α were unaffected.	Relative hypoxic, acute inflammation	Soft tissue trauma	7+7+7	In vivo study of tissue and blood samples in a rat model	2.5 ATA 3 days 2 hours/day
Sunkari et al 2014	Immunoblot, reporter gene assay and quantitative real time PCR for target gene expression	HBO_2 stabilized and activated HIF-1α and increased the HRE driven luciferase response as well as mRNA expression of target genes.	Relative hypoxic, chronic inflammation	Diabetic wounds	5	Ex vivo study of human dermal fibroblasts	2.5 ATA 1 hour
Yang et al. 2014	Immunohisto chemistry and western blot	HBO_2 reduced injury induced HIF-1α protein expression. Levels remained higher than healthy controls.	Hypoxic, acute inflammation	Induced traumatic brain injury	90	In vivo rat model	1.5 ATA 15 days 1,5 hours/day
Al Hadi et al. 2013	Real time-quantitativ-PCR and western blot	HBO_2 decreased disease mediated elevated levels of HIF-1α mRNA and protein expression. Levels were lower than normoxic controls.	Hypoxic, chronic inflammation	Osteonecrosis	Not specified	Ex vivo study human Peripheral Blood Monocytes	2.4 ATA 12 days 1,5 hours/day
Zhou et al. 2013	Quantitative PCR, Immunoflorescence and Western blot	HBO_2 decreased the disease mediated expression of HIF-1α mRNA and protein after 3 sessions, as compared to injured controls. Levels remained higher that healthy controls. No effect on healthy controls.	Relative hypoxic, acute inflammation	Animal model of spinal cord injury	40	In vivo rat model	2.0 ATA 2,6,10 or 17 days 2 x 1 hour/day the first 3 days then once/day
Chen et al. 2012	Real time-quantitative PCR	HBO_2 did not affect mRNA expressions levels in any of the tissue types.	Relative hypoxic, acute inflammation	Wound healing	5 + 5	In vivo mice model of wounds in skin and mucosa	2.5 ATA 2 or 4 days 2 x 2 hours/day (1 hour apart)
Sakata et al. 2010	Immunohisto chemistry	HBO_2 decreased HIF-1α protein expression compared to untreated controls.	Relative hypoxic, acute inflammation	Intraportal islet transplantation	2	In vivo study of liver islets from mouse model of diabetes	2.5 ATA 3 days 2 x 1 hour/day
Bai et al. 2009	Immunohisto chemetry and Western blotting	HBO_2 significantly reduced disease-induced HIF-1α proteins expression compared to diseased controls. HIF-1α remained higher than healthy controls.	Relative hypoxic, acute inflammation	Model of acute pancreatitis	10	In vivo rat model	2.6 ATA 1,5 hour
Milovanova et al. 2009	Western blot	HBO_2 increased HIF protein expression compared to controls in both blood and bone marrow.	Normoxic, non-inflammation	Vasculogenic stem cell growth	4	In vivo mouse model	2.8 ATA 2, 5 or 10 days 2 x 1,5 hours (12 hours apart)

Gu et al. 2008	Western blotting, HIF-1α DNA-binding, Real time-PCR.	HBO_2 increased HIF-1α protein expression and HIF-1α DNA-binding, as well as the HIF target gene EPO compared with to healthy controls.	Normoxic, non-inflammation	Rat focal cerebral ischemic model	5(western blot) 6 (DNA-binding)	In vivo rat model	2.0 ATA 5 treatments 1 hour every second day
Peng et al. 2008	Immunohistochemistry and western blot	HIF-1α protein expression was higher at all timepoints in HBO_2 group compared to controls, with maximum at day 3.	Normoxic, non-inflammation	Animal model of hypoxia	8	In vivo mouse model	2,5 ATA 2, 6 or 10 days 2 x 1 hour/day
Ren et al. 2008	Real time - QuantitativePCR, western blot and ELISA-based kit for DNA binding	No significant differences in HIF-1α mRNA expression, but HBO_2 increased the expression of HIF-1α protein and DNA binding in healthy rats compared to untreated controls.	Normoxic, non-inflammation	Model of liver regeneration after resection.	6 (PCR) 5 (DNA binding) 6 (western blot)	In vivo rat model	2.5 ATA 3 days 1 hour/day
Sun et al. 2008	Immunoblotting	HBO_2 reduced the injury-induced expression of HIF-1α protein compared to controls.	Hypoxic, acute inflammation	Stroke model with experimental cerebral ischemia	5	In vivo mouse model	3.0 ATA 95 min
Zhang et al. 2008	Western blot	HBO_2 decreased HIF-1α protein expression at day 7 compared to healthy controls. Levels were equally high between the two groups after 10 treatments.	Hypoxic, acute inflammation	Ischemic wound tissue	6	In vivo rat model	2.4 ATA 3, 7, 10 or 14 days 1,5 hours/day
Huang et al. 2007	Immunohistochemistry and western blot	HBO_2 decreased injury-induced elevated levels of HIF-1α protein expression. Levels remained higher than healthy controls.	Hypoxic, acute inflammation	Focal cerebral ischemia	8	In vivo rat model	2.5 ATA 2 hours
Calvert et al. 2006	Western blot	HBO2 significantly reduced HIF-1α protein expression compared to diseased controls. Levels remained higher than healthy controls.	Hypoxic, acute inflammation	Induced hypoxic ischemic cerebral insult	30	In vivo rat model	2.5 ATA 2 hours
Salhanick et al. 2006	DNA-binding Assay, Transient transfection assays	HBO_2 increased HIF-1α DNA binding activity, but not transcriptional activity of HIF target genes.	Relative hypoxic, acute inflammation	Acetaminophen poisoning	3	Ex vivo study of human hepatocytes	2.5 ATA 1,5 hours
Salhanick et al. 2006	Western blot	HBO2 upregulated disease-induced HIF-1α protein expression compared to controls.	Relative hypoxic, acute inflammation	Acetaminophen poisoning	3	In vivo mouse model	2.5 ATA 1,5 hours
Li et al. 2005	Immunohistochemistry and Western blotting	HBO_2 reduced the injury-mediatedhigh levels of HIF-1α protein expression at all time points compared to ischemic controls with a maximum at 96 hours. The level of HIF-1α remained higher than healthy controls.	Hypoxic, acute inflammation	Model of transient global cerebral Ischemia–hypotension	2(immunohistochemitry) 4(western blot)	In vivo rat model	3.0 ATA 2 hours

Ostrowski et al. 2005	Western blot, Immunohisto chemistry, and double fluorescent staining	HBO_2 decreased injury-induced HIF-1α proteins expression compared to injured controls. HIF-1α remained at the level of healthy controls.	Hypoxic, acute inflammation	Rat model of subarachnoid hemorrhage	14	In vivo rat model	2.8 ATA 2 hour

Table S2 – Studies on the effect of hyperbaric oxygen treatment on NF-κB

Study	Method	Outcome	O_2 Environment	Disease under study	No. of objects exposed to HBO_2	Specimen	HBO treatment
Syahrizal et al. 2022	Immuno-histo-chemistry	HBO_2 had no significant effect on NF-kB protein expression compared to disease-induced controls.	Relative hypoxic, chronic inflammation	Endometriosis	8	In vivo mouse model	2.4 ATA 10 days 1,5 hours/day (2 air-breaks)
Wang et al 2022	Immuno-flurescence-staining and western blot	HBO_2 decreased disease-induced elevated levels of NF-kB and NF-kB-p65 protein expression. Levels remained higher than health controls.	Relative hypoxic, chronic inflammation	Brain senescence	3	In vivo mouse model	2.0 ATA 14 days 1 hour/day
Chen et al. 2021	Immuno-fluorescence and Western blot	HBO_2 decreased injury-induced elevated levels of NF-kB protein expression. Levels remained higher than healthy controls.	Relative hypoxic, chronic inflammation	Burn injury	6	In vivo rat model	2.5 ATA 14 days 1,5 hours/day
Fratantonio et al. 2021	Western blot	HBO_2 significantly increased NF-kB protein expression 30 minutes and 3 hour after HBO_2 exposure. Levels were normalized after 24 hours.	Normoxic, non-inflammatory	Healthy subjects	4	In vivo study of human mononuclear cells	1.4 ATA 1 hour
Huang et al. 2020	Quantitative-PCR and Western blotting	Relative NF-kB mRNA and protein levels increased in response to HBO_2 compared to disease-induced controls.	Normoxic, acute inflammation	Diabetic model	3	Ex vivo human skin fibroblast	2.5 ATA 1, 2 or 3 days 1,5 hours/day
Huang et al. 2020	Quantitative-PCR and Western blotting	NF-kB mRNA and protein levels increased in response to HBO_2 compared to injured controls.	Relative hypoxic, chronic inflammation	Diabetic foot animal model	3	In vivo mouse model	2.0 ATA 30 days 1 hour/day
Ko et al. 2020	Immunohis-tochemical and immuno-fluorescent staining and western blot	HBO_2 decreased disease-induced elevated levels of NF-kB protein expression. Levels remained higher than healthy controls.	Hypoxic, acute inflammation	Acute renal ischemia-reperfusion injury	10 +10	In vivo rat model	2.4 ATA 3 days 1,5 hours/day
Liang et al. 2020	Real time-PCR, immunohisto-chemistry and Western blot	HBO_2 decreased injury-induced elevated levels of NF-kB mRNA and protein expression in the 1.6 ATA treatment group when applied until 6 hours after the trauma.	Relative hypoxic, acute inflammation	Traumatic brain injury	30 or 30	In vivo rat model	1.6 ATA or 2.2 ATA 14 days 1 hour/day
Anguiano-Hernandez et al 2019	Immuno-histo-chemistry	Insignificant decrease in NF-kB protein expression compared to before HBO2. No control group.	Relative hypoxic, chronic inflammation	Diabetic foot ulcers	36	In vivo study of human wound tissue	1.4 ATA 20 days 1,5 hours/day
Sun et al. 2019	ELISA	HBO2 decreased NF-kB protein expression after 3 to 30 days.	Relative hypoxic, acute inflammation	Spinal cord injury	41	In vivo study of human plasma	2.0 ATA 1, 3, 7, 10 or 30 days 1 hour/day

Song et al. 2018	Immuno-histo-chemistry	NF-kB protein expression decreased in response to HBO_2 compared to controls.	Relative hypoxic, chronic inflammation	Keloid tissue	8	In vivo study of human tissue	2.0 ATA 20 days or more 2 hours/day
Lu et al. 2016	Immuno-histo-chemistry and western blot	NF-kB protein expression decreased in response to HBO_2 in both chemotherapy treated, and untreated glioma mice compared to their respective controls.	Relative hypoxic, chronic inflammation	Healthy mice inoculated with human glioma cells	5 (glioma mice) and 5 (Nimustine treated glioma mice)	In vivo mouse model	2.5 ATA 21 days 1,5 hours/day
Meng et al. 2016	Western blot	HBO_2 decreased injury-induced elevated levels of NF-kB-p65 protein expression compared to controls. Levels remained higher than healthy controls.	Relative hypoxic, acute inflammation	Secondary brain injury after trauma	10	In vivo rat model	1.2 ATA 2 sessions 2 x 1 hours (10 hours apart)
Kang et al 2015	ELISA	HBO_2 decreased injury-induced elevated levels of NF-kB protein expression after 2 to 7 days of treatment. Levels remained higher than healthy controls.	Relative hypoxic, acute inflammation	Spinal cord injury	6 in each group	In vivo rat model	2.5 ATA 1, 2, 3, 7 or 14 days 1 x 45 min/day
Tan et al. 2014	Real time PCR and western blot	HBO_2 decreased injury-induced elevated levels of NF-kB-p65 protein expression after 1 to 7 days of treatment.	Relative hypoxic, acute inflammation	Spinal cord injury	6 in each group	In vivo rat model	2.0 ATA 1, 2, 3, 7 or 14 days 1 hour/day
Liang et al 2013	Immuno-histo-chemistry and western blot	HBO_2 decreased injury-induced elevated levels of NF-kB protein expression. Levels remained higher than healthy controls.	Hypoxic, acute inflammation	Epigastric skin flap grafting	7 (6 sessions) or 7 (8 sessions)	In vivo rat model	2.0 ATA 6 or 8 days 2 x 1 hour for 3 days, then 1 hour for 2 days
Yang et al 2013	Real time-PCR, immuno-histo-chemical staining and western blot	HBO2 decreased injury-induced elevated levels of NF-kB mRNA and protein expression levels at day 3, 7, and 14.	Relative hypoxic, acute inflammation	Spinal cord injury	6-10 per group	In vivo rat model	2.5 ATA 2, 6, 10 or 17 days 2 x 45 min for 3 days, thereafter 45 min/day.
Madden et al. 2011	ELISA	HBO_2 increased NF-kB protein expression after 4 hours in comparison to before HBO_2.	Normoxic, non-inflammatory	Healthy humans.	10	In vivo study of human peripheral blood mononuclear cells	2.8 ATA 1 hour
Yu et al. 2009	Electro-phoretic mobility shift assay	HBO_2 decreased the DNA binding activity of NF-kB compared to disease-induced controls. Levels remained higher than healthy controls.	Relative hypoxic, acute inflammation	Acute pancreatitis	40	In vivo study of rat peripheral blood	2.4 ATA (90% O_2) 1 session of 1, 3, 5 or 7 hours
Sakoda et al. 2004	Immuno-histo-chemistry	HBO_2 decreased the mucosal nuclear factor-B activation within 4–6 hours after LPS stimulation.	Relative hypoxic, acute inflammation	Intestinal mucosal injury	1	In vivo Lipopolysacc harid rat model	2.5 ATA 1 hour
